# Health care utilization following “digi-physical” assessment compared to physical assessment for infectious symptoms in primary care

**DOI:** 10.1186/s12875-021-01618-2

**Published:** 2022-01-12

**Authors:** Artin Entezarjou, Maria Sjöbeck, Patrik Midlöv, Veronica Milos Nymberg, Lina Vigren, Ashkan Labaf, Ulf Jakobsson, Susanna Calling

**Affiliations:** 1 Department of Clinical Sciences in Malmö/Family Medicine, Center for Primary Health Care Research, Lund University, Box 50332, 20213 Malmö, Sweden; 2 Capio Go, Gothenburg, Sweden; 3 Department of Clinical Sciences, Lund University, Lund, Sweden

**Keywords:** Telehealth, Telemedicine, eVisits, Primary care, Utilization, Infection

## Abstract

**Background:**

The use of chat-based digital visits (eVisits) to assess infectious symptoms in primary care is rapidly increasing. The “digi-physical” model of care uses eVisits as the first line of assessment while assuming a certain proportion of patients will inevitably need to be further assessed through urgent physical examination within 48 h. It is unclear to what extent this approach can mitigate physical visits compared to assessing patients directly using office visits.

**Methods:**

This pre-COVID-19-pandemic observational study followed up “digi-physical” eVisit patients (*n* = 1188) compared to office visit patients (*n* = 599) with respiratory or urinary symptoms. Index visits occurred between March 30th 2016 and March 29th 2019. The primary outcome was subsequent physical visits to physicians within two weeks using registry data from Skåne county, Sweden (Region Skånes Vårddatabas, RSVD).

**Results:**

No significant differences in subsequent physical visits within two weeks (excluding the first 48 h) were noted following “digi-physical” care compared to office visits (179 (18.0%) vs. 102 (17.6%), *P* = .854). As part of the “digital-physical” concept, a significantly larger proportion of eVisit patients had a physical visit within 48 h compared to corresponding office visit patients (191 (16.1%) vs. 19 (3.2%), *P* < .001), with 150 (78.5%) of these eVisit patients recommended some form of follow-up by the eVisit physician.

**Conclusions:**

Most eVisit patients (68.9%) with respiratory and urinary symptoms have no subsequent physical visits. Beyond an unavoidable portion of patients requiring urgent physical examination within 48 h, “digi-physical” management of respiratory and urinary symptoms results in comparable subsequent health care utilization compared to office visits. eVisit providers may need to optimize use of resources to minimize the proportion of patients being assessed both digitally and physically within 48 h as part of the “digi-physical” concept.

**Trial registration:**

Clinicaltrials.gov identifier: NCT03474887.

**Supplementary Information:**

The online version contains supplementary material available at 10.1186/s12875-021-01618-2.

## Background

Utilization of digital primary care visits is rapidly increasing [1] for various clinical issues, including assessment of respiratory or urinary symptoms [2–4]. While synchronous video-based visits (virtual visits) are a commonly used format, asynchronous chat-based visits (eVisits) offer a novel approach where multiple patients can be assessed simultaneously [5]. Of the 13 digital primary care health care providers reviewed by The Swedish Health and Social Care Inspectorate [6], seven include asynchronous text-based communication. Unlike virtual visits or phone consultations, eVisits also allow staff to conduct other tasks at their primary health care center while awaiting patient response, as well as to consult colleagues more seamlessly when needed before responding to patients. Unlike portal messaging, eVisits usually offer an infrastructure that allows for more rapid “live” text chats with automated questionnaires usually integrated prior to the chat commencing.

In Sweden, the government is currently adopting a national vision of achieving good and equal health and welfare by 2025 by becoming the world leader at using digitization and eHealth [7]. Swedish primary care is almost entirely publicly funded by 21 regions, with each region deciding which information technology systems to implement. Each region has public primary health care centers, but regions also reimburse private health care providers for primary care services using various combinations of capitation and pay-per-service. The emergence of several private eVisit providers, billing regions for digital-only primary care services, has been reported to further fragment Swedish primary care, and better integration between eVisits and physical care has been recommended to move towards the national eHealth vision for 2025 [7]. Subsequently, all 21 regions have now developed their own digital primary care platforms. This is in addition to the private digital-only providers, which offer their services nationally.

It is unclear to what extent eVisits can successfully replace office visits for the assessment of infectious symptoms. Using eVisits may improve patient access to care [3], be time-saving [8] and maintain high patient satisfaction [9] while reducing risk of, e.g. transmission of COVID-19 during the pandemic [10]. eVisits may also allow primary care staff to work remotely to a larger extent and harness a more flexible working environment. Finally, cost-savings per episode of care may be realized [2, 4, 11], and knowing which patients are likely to require further physical follow-up after an eVisit may help health care providers decide what clinical issues to directly assess using an office visit.

An emerging strategy, which has been suggested by recent qualitative work, is to maximize the utilization of eVisits where possible, focusing on a “digi-physical” approach where the patient is initially assessed via an eVisit with the possibility to schedule continued management with a physical examination when needed [5]. Previous studies on healthcare utilization following eVisits for minor acute symptoms, including cough [12] and upper respiratory tract symptoms [13], found that roughly two-thirds of patients had their concerns resolved without further interactions with the health care system. Studies comparing eVisits to office visits found either no significant differences [4] or higher [12, 14] rates of subsequent health care contacts following eVisits. Given these inconsistent results in the dawn of increasing eVisit utilization, further studies are needed to investigate subsequent health care utilization following eVisits compared to office visits [3]. Respiratory symptoms have been described as one of the most common chief complaints among eVisit users [12].

The aim of this study was to investigate whether there were any differences in the frequency of healthcare contacts following initial management of respiratory or urinary symptoms using traditional office visits compared to “digi-physical” management. We define “digi-physical” management as patients having their initial clinical encounter through an eVisit, with urgent physical care within 48 h when needed.

## Methods

### Setting and population

This observational study compared patients residing in the Skåne region, Sweden’s third largest county with 1.4 million inhabitants. Patients were managed using “digi-physical” care or using traditional office visits at 16 primary health care centers across Skåne. Apart from the previously mentioned digital primary care providers, patients have the option to seek physical care at their primary health care center, which is usually open between 8 a.m. and 5 p.m. Patients can also seek care at out of hours clinics, open from 5 p.m. to 9 p.m., or visit the emergency department of any hospital. All index visits in the current study were conducted at Capio, one of Sweden’s largest primary health care center providers, which has adopted the “digi-physical” model since May 2017, using an eVisit platform developed by Doctrin AB. ﻿At the time of the study, Capio was the only known primary health care provider that offered both office visits and eVisits, while other eVisit providers simply referred patients who were deemed to require a physical examination. This meant the patient and physician had to restart the consultation, which resulted in two payments.

Inclusion criteria were visits with a chief complaint of sore throat, cough, cold/flu-symptoms or urinary symptoms as specified by free-form text, or visits with a documented International Classification of Disease code J030 (streptococcal tonsillitis), J069 (acute upper respiratory infection), or N300 (cystitis) [15]. Index visits were selected by identifying each patient’s earliest dated physician visit (for the chief complaints included) between March 30th, 2016 and March 29th, 2017 (office visits only) or between March 30th, 2018 and March 29th, 2019 (eVisits and office visits), i.e. before the COVID-19 pandemic. Exclusion criteria were patients aged < 18 years, residence outside of Skåne county, male patients with urinary symptoms and identifiable visits for similar chief complaints in the past 21 days. In addition to this, each patient was only allowed to contribute with one index-visit across the entire cohort. The earliest dated visit was chosen as the included index visit.

### The platform

The eVisit platform assessed in this study can be accessed by patients through their smartphone, computer, or tablet seven days a week from 7 a.m. to 10 p.m. Patients choose their chief complaint and proceed to answer a set of symptom-specific questions. Answers are structured in a report presented to a physician who then initiates a two-way text-based communication within 15 min for medical decision-making, including staying available for observation (watchful awaiting) or utilizing “digi-physical” care by scheduling a physician appointment at a physical Capio primary health care center within 48 h if needed. The receiving physician at the primary health care center gets access to the same medical history generated by the eVisit platform and the text from the chat communication between the physician and the patient for an improved transition. Capio has protocols for each chief complaint, with indications for scheduling physical care and key performance indicators to follow-up protocol adherence.

### Power calculation and recruitment

**Fig. 1:**
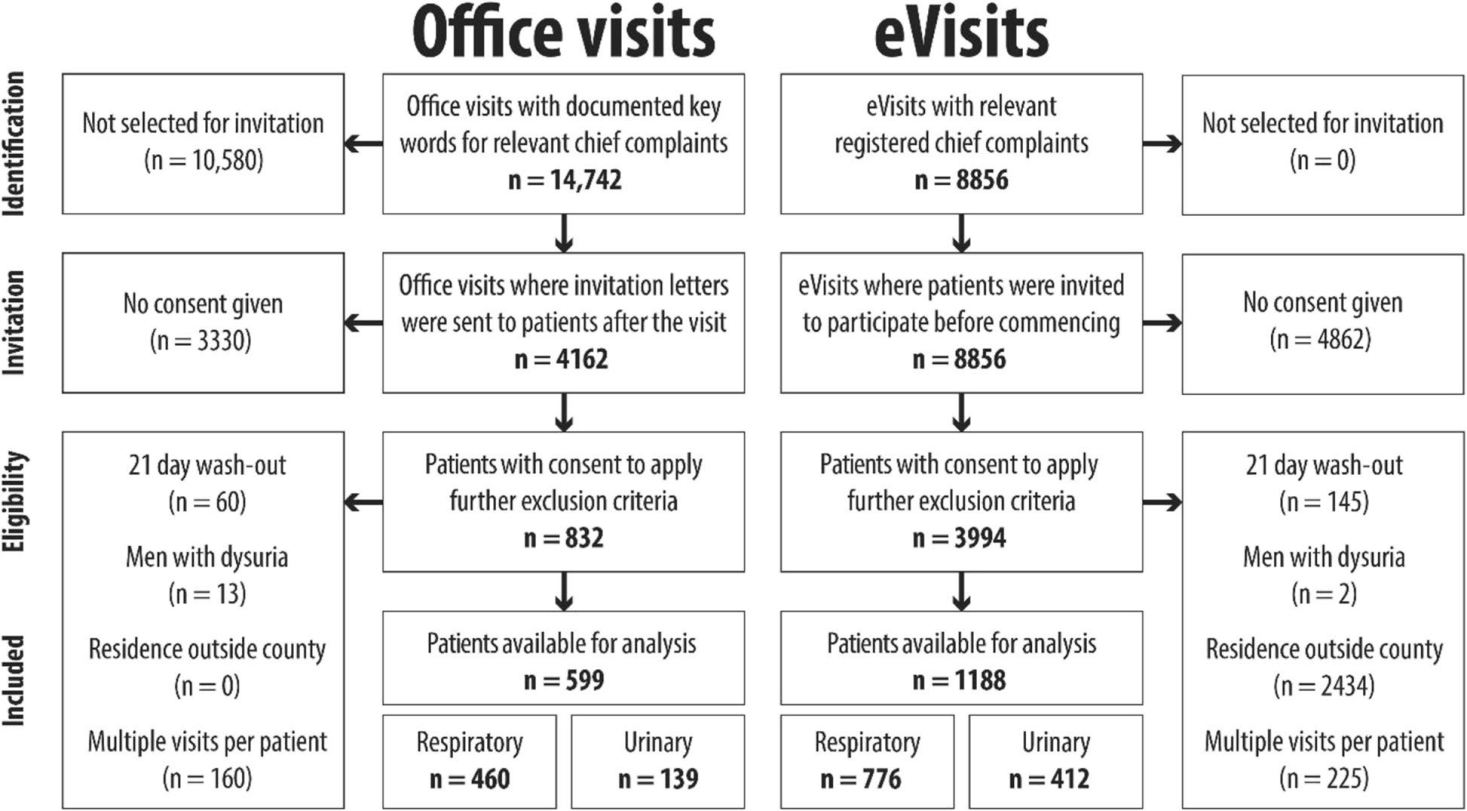
Flow-chart of patient recruitment

Previous research on office visits for upper respiratory tract symptoms reported a 26% two-week follow-up rate [16]. Using a binary outcome power calculation with a non-inferiority limit of 6.5%, an alpha level of 0.05, for 80% power, we estimated needing 564 visits per group. Informed consent was acquired from all included participants. eVisit patients were invited once and consented digitally prior to their visit. For office visit patients, data extraction software (by Medrave Software AB) was used to identify adult patients with key words in the electronic medical records free-form text corresponding to included chief complaints (Additional file 1). A random selection of identified office visit patients were invited through letters, including two reminders to non-responders, posted to their home address after their visit with a signed response returned in a prepaid envelope as previously described [15]. After acquired consent, remaining exclusion criteria were applied resulting in the final cohort (Fig. 1).

### Data collection

Baseline data including chief complaint, visit date, age, sex, and patient residence were acquired from the medical record of the healthcare provider using the same data extraction software that identified patients. Automatically extracted data on chief complaints had previously been manually validated by reading all free-form text in the electronic medical record of the index visit for a subset of visits (*n* = 783) [15]. For eVisits only, data were also extracted regarding recommended follow-up by the physician as either self-care, continued eVisit, or recommended outpatient physical visit (urgent or non-urgent) as this was documented as part of the eVisit electronic medical record template. Patient data related to county-wide health care contacts within two weeks of their index visit were acquired from a county-wide registry (Region Skånes Vårddatabas, RSVD) registering all health care contacts billed to the local county council, including set diagnoses and health unit names for each health care contact. The database does not include visits provided through health care providers without a reimbursement contract with the local county council, but such visits only account for around 1% of all healthcare expenditure in Sweden [1].

The primary outcome was proportion of patients with one or more physical visits to a physician within two weeks after the first 48 h of their index visit, as “digi-physical care” per definition involves a proportion of visits inevitably proceeding to physical examination within 48 h of their eVisit assessment. Visits beyond 48 h after index thus represent visits not expected in the “digi-physical” model. To make subsequent utilization beyond this window was comparable to office visits, we excluded physical visits within 48 h of the index visit after both eVisits and office visits in the primary outcome. As most patient-initiated primary care contacts in Swedish primary care are initially managed through nurse telephone triage, the number of outpatient telephone contacts with nurses within two weeks of the index visit (not including the day of the index visit) was evaluated as a secondary outcome. Other secondary outcomes included proportion of additional outpatient physical visits within 48 h of index visit, visit location (primary care, out-of-hours visit, emergency department, or other outpatient clinic), and proportion of patients admitted for inpatient care.

For eVisit patients only, we also calculated secondary outcomes regarding proportions of index visits in which the patient was recommended self-care, continued digital care or physical follow-up, respectively. eVisit-physician-documented recommendation for an urgent visit within 48 h, a non-urgent primary care visit, and referral to other healthcare providers (including emergency departments) were all considered a physician recommendation for physical follow-up. In 13 cases where data regarding recommended follow-up were missing, data were manually collected through review of electronic medical records.

### Statistical analyses

Analysis was conducted in IBM SPSS version 26. Visits with a chief complaint of sore throat, cough, and common cold/influenza were all grouped together to a “respiratory” group, while visits for urinary symptoms were considered a separate group.

Student’s t-tests were used to compare continuous data and were presented with mean and standard deviation. Chi-square test was used to compare categorical data, presented with percentage.

We hypothesized that there was no clinically relevant difference in the number of physical visits within two weeks when comparing eVisit patients to office visit patients, excluding the first 48 h where a larger portion of eVisits patients are expected to be encouraged to proceed to a physical visit. Hypothesis testing was conducted by comparing patients with index eVisits and index office visits, after excluding patients with subsequent physical visits within 48 h.

Sensitivity analyses were conducted comparing subsequent physical visits including visits within 48 h, but instead excluding eVisit patients recommended various levels of physical follow-up to evaluate robustness of findings.

As chief complaint and age may confound risk of further follow-up, multiple binary logistic regressions were conducted with physical visit or nurse phone contact as the dependent variable and visit type as the independent variable. Office visits were used as the reference group, with the enter regression models adjusted for age and chief complaint.

Exploratory subgroup analyses were conducted to evaluate health care utilization of eVisit patients who received various follow-up recommendations.

Further subgroup analyses were conducted to calculate the proportion of physical visits within various levels of care (ranked from highest to lowest acuity: emergency care, out of hours care (including ambulatory care), primary care, and other outpatient care) during the follow-up period. For patients in contact with multiple levels of care, the highest level of care was included.

Physical visit locations classified as emergency or other outpatient care were manually reviewed by looking up health unit names of the health care contacts as specified in RSVD to make sure the visit location was validly classified. For both groups, inpatient care within the entire follow-up period was also compared.

For a subset of patients with physical visits within two weeks (836 respiratory and 434 urinary complaints), the first three diagnoses recorded in the electronic medical record were manually reviewed together with a specialist in family medicine (SC and PM) and used to assess whether the visit was likely related to or unrelated to the index-visit.

The study was registered at clinicaltrials.gov (Identifier: NCT03474887) and reported using the STROBE-checklist.

## Results

### Baseline demographics

Among office visit patients, there were significantly more visits for respiratory symptoms and significantly fewer visits for urinary symptoms compared to eVisit patients. Office visit patients were also significantly older than eVisit patients. No differences in sex distribution were noted (Table 1).

**Table 1:** Baseline demographics

	eVisit patients (n = 1188)	Office visit patients (n = 599)	*P*-value for difference
**Respiratory chief complaint, n (%)**	776 (65.3%)	460 (76.8%)	< 001^a^
**Urinary chief complaint, n (%)**	412 (34.7%)	139 (23.2%)	< 001^a^
**Age, mean (std dev)**	41.3 (14.4)	52.5 (19.0)	< 001^b^
**Sex, n (% women)**	924 (77.8%)	432 (74.2%)	.097^a^

^a^Chi-square test

^b^Student’s t-tests

### Physical visits within two weeks

There were no significant differences in proportion of physical visits after the first 48 h but within two weeks of the index visit when comparing eVisit patients to office visit patients (18.0% vs. 17.6%, *P* = .854). Within 48 h of the index visit, a larger proportion of eVisit patients had a physical visit compared to office visit patients (16.1% vs. 3.2%, *P* < .001). Results were robust to subgroup analyses of each chief complaint as well as after adjusting for age and chief complaint in logistic regression analyses (Table 2). Considering all 1188 eVisit patients, a total of 818 (68.9%) had no physical visit within the entire follow-up period. Sensitivity analyses including all physical visits within two weeks of the index visit demonstrated similar results once eVisit patients recommended follow-up were excluded. Two-week physical visit rates, including the first 48 h, were significantly higher comparing all eVisit patients to office visit patients (370 (31.1%) vs. 123 (20.5%), *P* < .001), but no significant difference remained when excluding eVisit patients recommended primary care follow-up within 48 h (215 (21.5%) vs. 123 (20.5%), *P* = .640). When excluding eVisit patients recommended any form of physical follow-up (both urgent and non-urgent), two-week physical visit rates were 181 (19.1%) vs. 123 (20.5%), *P* = .475, for eVisit patients vs. office visit patients, respectively.

**Table 2:** Health care contacts within two weeks and regression models for office visit patients compared to eVisit patients

	eVisit patients (*n* = 1188)	Office visit patients (*n* = 599)	*P*-value
**Physical visit within two weeks, n (%)**^a^	179 (18.0%)	102 (17.6%)	.854^f^
** Following respiratory symptoms, n (%)**^b^	126 (20.0%)	77 (17.4%)	.294^f^
** Following urinary symptoms, n (%)**^b^	53 (14.5%)	25 (18.1%)	.314^f^
** Odds ratio**^c^ **for eVisit patients compared to office visit patients (95% CI)**	1.24 (0.92-1.66)^h^		.152^g^
**Physical visit within 48 h, n (%)**	191 (16.1%)	19 (3.2%)	< .001^f^
** Following respiratory symptoms, n (%)**^d^	145 (18.7%)	18 (3.9%)	< .001^f^
** Following urinary symptoms, n (%)**^d^	46 (11.2%)	1 (0.7%)	< .001^f^
**Nurse telephone contact within two weeks, n (%)**^e^	101 (8.5%)	50 (8.3%)	.912^f^
** Following respiratory symptoms, n (%)**^d^	66 (8.5%)	36 (7.8%)	.675^f^
** Following urinary symptoms, n (%)**^d^	35 (8.5%)	14 (10.1%)	.527^f^
** Odds ratio**^c^ **for eVisit patients compared to office visit patients (95% CI)**	1.04 (0.71-1.52)^i^		.842^g^

^a^Not including physical visits within 48 h of index visit. *n* = 997 eVisit patients and *n* = 580 office visit patients

^b^Percentages refer to patients with the specified chief complaint, excluding patients with a physical visit within 48 h of their index visit. For respiratory eVisit patients *n* = 631 and office visit patients *n* = 442. For urinary eVisit patients *n* = 336 and office visit patients *n* = 138

^c^Each regression model was adjusted for age and chief complaint

^d^Percentages refer to patients with the specified chief complaint. For respiratory eVisit patients *n* = 776 and office visit patients *n* = 460. For urinary eVisit patients *n* = 412 and office visit patients *n* = 139

^e^Not including the index visit date

^f^Chi-square test

^g^Logistic regression

^h^Nagelkerke R Square: 0.0127

^i^Nagelkerke R Square: 0.0008

### Nurse telephone contacts within two weeks

No significant differences in nurse telephone contacts within two weeks following the index visit were noted between eVisit patients and office visit patients. Results were robust to subgroup analyses of each chief complaint as well as after adjusting for age and chief complaint in logistic regression analyses (Table 2).

### Recommended follow-up for eVisit patients

Analysis of the 191 (16.1%) eVisit patients with a physical visit within 48 h showed that 150 (78.5%) had been recommended some form of follow-up by the eVisit physician, including 107 (56.0%) specifically recommended a physical follow-up within 48 h, 28 (14.7%) recommended non-urgent physical follow-up, and 15 (7.9%) recommended a follow-up eVisit.

818 eVisit patients (68.9%) were recommended self-care or no follow-up. Among these, the number of patients who had a physical visit within two weeks, including the index visit date, was 144 (17.6%).

132 eVisit patients (11.1%) were recommended follow-up with an additional eVisit. Among these, the number of patients who had a physical visit within two weeks, including the index visit date, was 37 (28.0%).

238 eVisit patients (20.0%) were recommended some form of physical follow-up. Among these, the number of patients who had a physical visit within two weeks, including the index visit date, was 189 (79.4%).

Among the 238 patients recommended physical follow-up, 163 eVisit patients (68.4% of patients recommended physical follow-up, 13.7% of all eVisit patients) were recommended physical follow-up within 48 h. Among these, the number of patients who had a physical visit within 48 h was 107 (65.6%).

### Level of care and unit

Within two weeks of the index visit, most subsequent physical visits during the follow-up period occurred at primary health care centers. Sixteen patients were admitted for inpatient care during the entire follow-up period, with no significant differences noted between eVisit and office visit patients (Table 3).

**Table 3:** Level of care of physical visits between 48 h and two weeks

	eVisit patients (*n* = 1188)	Office visit patients (*n* = 599)	*P*-value for difference
Physical visit within two weeks, n (%)*	179 (18.0%)	102 (17.6%)	.854^a^
** Of which primary care**	128 (71.5%)	73 (71.6%)	N/A
** Of which out of hours care**	15 (8.4%)	2 (2.0%)	N/A
** Of which emergency care**	14 (7.8%)	12 (11.8%)	N/A
** Of which other outpatient care**	22 (12.3%)	15 (14.7%)	N/A
**Physical visit within 48 h, n (%)**	191 (16.1%)	19 (3.2%)	< .001^a^
** Of which primary care**	150 (78.5%)	6 (31.6%)	N/A
** Of which out of hours care**	27 (14.1%)	0 (0.0%)	N/A
** Of which emergency care**	11 (5.8%)	10 (52.6%)	N/A
** Of which other outpatient care**	3 (1.6%)	3 (15.8%)	N/A
**Admitted within entire follow-up period, n (%)**	8 (0.7%)	8 (1.3%)	0.161^a^

*Not including physical visits within 48 h of index visit. *n* = 997 for eVisit patients and *n* = 580 for office visit patients

^a^Chi-square

## Discussion

### Principal results

After 48 h, no differences were found in subsequent physical visits within two weeks for eVisit patients compared to office visit patients. The results persisted when adjusted for age and chief complaint. Within the 48-h timeframe, a larger proportion of eVisit patients had a physical visit, 78.5% of which were recommended some form of follow-up as part of the health care provider’s protocol for safe “digi-physical” management. Considering all eVisit patients, 68.9% concluded their eVisit without additional physical visits within two weeks.

### Strengths and limitations

Results should be interpreted with consideration for several limitations. As randomization was not performed, groups may differ regarding comorbidities, symptom severity and previous health care contacts. The office visit group may, for instance, represent patients seeking care after referral from other healthcare providers, including digital ones, while eVisit patients might be seeking care earlier in their symptom development. This was addressed to the extent possible by excluding previously identified healthcare contacts, including each patient only once across all groups and adjusted regression analyses.

eVisit patients were recruited prospectively before the visit commenced, while office visit patients were recruited retrospectively weeks to months after their visit. The inclusion method might have led to inclusion bias and is therefore a limitation of this study.

No reliable data were available regarding subsequent digital care contacts, including eVisits and virtual visits to the current and other health care providers. Non-physician visits to other physical units such as midwife offices and youth clinics also represent additional subsequent health care utilization not included in the current study thus limiting conclusions regarding total healthcare utilization. It is also uncertain to what extent physical visits were planned provider-initiated or unplanned patient-initiated.

The results of the current study cannot be generalized as they are specific to the context of “digi-physical” care with the specific eVisit platform used by the current healthcare provider. The current sample size is not large enough to detect clinically meaningful differences in emergency department visits or hospital admissions, and all secondary findings should be interpreted with caution.

Nonetheless, the study also has several strengths. To the best of our knowledge, this is the first study comparing the trajectory of “digi-physical” care with traditional primary care office visits based on chief complaint, using index visits from the same healthcare provider. Comprehensive data were available on subsequent health care utilization due to the public health care system in Sweden. No data were missing in the final analysis. Data were manually evaluated and validated via a manual review of electronic health records. Separating visits within 48 h as a part of the “digi-physical” model adds a new dimension to the existing literature of follow-up after eVisits compared to office visits as heterogeneity in clinical presentation means that a portion of eVisit patients inevitably will need to proceed to physical examination as part of the same clinical episode.

An alternative interpretation of our data may be that all subsequent visits after the index visit, including those within 48 h, should be part of the primary outcome as each visit involves a new clinical encounter. However, results from such an analysis would not provide meaningful insights into subsequent utilization after those who need urgent physical examination have been assessed. Results were also robust to sensitivity analyses excluding eVisit patients recommended primary care follow-up within 48 h. The choice of 48 h as the landmark for this distinction, however, may be arbitrary and 24 h or 72 h may be equally relevant.

### Physical visits within two weeks

The current eVisit platform differs from traditional direct-to-consumer telemedicine where providers need to refer or recommend patients to seek physical care at their own primary health care center. Here, physical visits could be scheduled to the same health care provider with the automated medical history and chat forwarded accordingly. However, at the time of the study, eVisit physicians usually did not schedule a physical follow-up to themselves. Thus, a second physician once again needed to assess the previous medical history and chat conversation prior to the physical examination. The results of the study may have been different had there been full physician continuity in the “digi-physical” model, since continuity influences health care utilization [5, 17]. We speculate that “digi-physical” management may be made more efficient by allowing for the same eVisit physician to follow-up with a physical visit when needed (“person-level” continuity) rather than a separate physician within the same organization (“provider-level” continuity). Results are also specific to the included chief complaints, which are relatively uncomplicated. Further research is needed to evaluate other chief complaints relevant to primary care, such as routine diabetes follow-ups or psychiatric assessment. Qualitative data suggests that the eVisit platform, may not be optimal for management of more complex clinical issues [5]. While almost 70% of eVisit patients had no additional physical visit within two weeks, it is unclear whether the included eVisits represent substitutions to physical primary care visits, or new utilization due to ease of access to eVisits [18].

After 48 h, visits were more likely patient-initiated as the provider had no protocols for physician-initiated follow-up beyond 48 h. The similar rate of follow-up suggests that initial “digi-physical” management in this cohort successfully concluded visits similarly to initial management using an office visit, although the study was not powered to assess possible differences at the various levels of care. Furthermore, the lack of significant differences in nurse telephone contacts following the date of the index visit suggests that patients do not contact their primary health care center more often after an eVisit compared to office visits. The lower percentage of subsequent nurse telephone contacts within the follow-up period compared to the proportion of primary care physical visits may be explained by “digi-physical” scheduling bypassing nurse telephone triage.

Within 48 h, a greater proportion of patients assessed through eVisits had a subsequent physical visit compared to patients initially assessed through office visits. This disparity reflects the “digi-physical” model of care with protocols requiring eVisit physicians to schedule certain patients, such as those reporting severe dyspnea, for physical follow-up compared to traditional office-based care without such protocols. As eVisit protocols are new and heterogenous when comparing various health care providers, future research should compare and evaluate various protocols over time to find the optimal protocol for safe and cost-effective eVisit management. This includes identifying and defining red flag symptoms such as fever associated with respiratory symptoms.

Manual evaluation of diagnoses recorded on subsequent physical visits within two weeks suggested that most visits were related to the index visit. Physicians may be reluctant to assess red flags indicating possible severe infections in the eVisit setting [5]. “Double” physician assessment following eVisits may raise concerns regarding cost-effectiveness and misuse of physician resources. In Sweden, patients are often initially assessed by triage nurses, which may here represent an alternative solution to apply protocols without physician resources. Unless subsequent visits are made more efficient by the prior digital patient history, as suggested by qualitative research [5], certain chief complaints may be better managed with the traditional model of care. This remains to be elucidated by future research.

No novel findings emerged when exploring each chief complaint separately. For urinary symptoms, “digi-physical” management may represent an alternative to current practice as current guidelines also support management of uncomplicated urinary tract infections without a physical examination [19] and is consistent with previous research that found no differences in antibiotic prescription rates when comparing eVisits and office visits for dysuria [15].

Considering respiratory symptoms, the current findings are in-line with previous research that found higher follow-up rates within 24 and 48 h of telemedicine visits for adult sinusitis [20] and pediatric acute respiratory infections [14]. One American study, with a large, matched population, also noted higher follow-up rates both within 48 h and within three weeks for acute respiratory infections [21]. Two-thirds of respiratory eVisits had no additional visits within two weeks; this is in-line with predictions made after review of primary care electronic medical records [22] as well as previous studies on eVisits [13].

Longer-term studies found lower [16, 23] or no differences [2, 4, 24, 25] in follow-up rates up to three weeks after telemedicine visits for various acute conditions. Some of these studies included telemedicine follow-up in their outcomes [4, 13, 16, 23, 24], while the current study did not. Lower follow-up rates after telemedicine in some of these studies may also be explained by eVisit providers unable to schedule follow-ups, as opposed to the current study with a low barrier to scheduling follow-up appointments within the same healthcare provider when needed.

### Recommendation and level of care for eVisits

There is a trend where a “higher level” of recommended follow-up by eVisit physicians is reflected in a larger proportion of patients having a subsequent visit within the entire follow-up period. Even though 370 (31.1%) of eVisits were recommended some form of follow-up (both digitally or physically) and 370 patients (31.1%) had a physical visit within two weeks, physician recommendations were not always in-line with patient healthcare utilization. “Patient adherence” was 79.4% for recommended physical follow-up, and 82.4% for recommended self-care/no follow-up. Previous research on physician triage based on digital patient histories suggests high inter- and intra-rater variability in primary care triage thus making it difficult to optimize this process [26].

### Implications for the national eVisit strategy

The results encourage the use of the “digi-physical” approach as congruent with the national eHealth vision for 2025 [7] from an efficiency standpoint as patients, health care providers and regions can resolve a larger portion of medical issues using the “digi-physical” approach without additional subsequent health care contacts. From an access and equality standpoint, however, more research is needed as barriers remain for eVisit use by all segments of the population, such as those with foreign languages, low digital literacy or other disabilities [27].

## Conclusion

“Digi-physical” management of respiratory and urinary symptoms in the context of the currently studied eVisit platform results in similar utilization of physical visits within two weeks compared to initial management using traditional office visits. Future research should explore time consumption of scheduled “digi-physical” visits with and without physician continuity. A significantly larger proportion of eVisit patients had a physical visit within 48 h, often having been recommended follow-up by their eVisit physician, compared to corresponding office visit patients. As such, future research may need to explore which clinical issues to refer directly for physical assessment, as well as evaluate the effects of continuity on “digi-physical” utilization.

## Supplementary Information

**Additional file 1.** Key words used by automatic data extraction software for identification of patients with relevant chief complaints for recruitment. Terms were chosen based on clinical experience and reported phrases commonly used according to primary health care staff as reported by primary health care center managers. Key words were not used as part of strings such that the entire phrase had to be present in order for patients to be identified.

## Data Availability

The datasets used and/or analyzed during the current study are available from the corresponding author on reasonable request up to ten years following publication.
