# Supplementary material for: Health care utilization following “digi-physical” assessment compared to physical assessment for infectious symptoms in primary care
Source: BMC Prim Care. 2022 Jan 12;23:4. doi: 10.1186/s12875-021-01618-2 (PMC8753946; doi:10.1186/s12875-021-01618-2)
Supplement: Supplementary file 1 — Additional file 1. Key words used by automatic data extraction software for identification of patients with relevant chief complaints for recruitment. Terms were chosen based on clinical experience and reported phrases commonly used according to primary health care staff as reported by primary health care center managers. Key words were not used as part of strings such that the entire phrase had to be present in order for patients to be identified. [file 12875_2021_1618_MOESM1_ESM.docx]

Appendix 1: Key words used by automatic data extraction software for identification of patients with relevant chief complaints for recruitment. Terms were chosen based on clinical experience and reported phrases commonly used according to primary health care staff as reported by primary health care center managers. Key words were not used as part of strings such that the entire phrase had to be present in order for patients to be identified.

|  | Swedish text  (used in the current study) | English translation |
| --- | --- | --- |
| **Sore throat** | Ont i halsen, halsont, hals ont, halssmärta, hals smärta, halssmärtor, hals smärtor, tonsillit, halsfluss  hals fluss, svalg | Sore in throat, sore throat, throat pain, throat pains, tonsilitis, strep throat, throat |
| **Urinary symptoms** | Miktionsbesvär, miktions besvär, miktionssvårigheter, miktions svårigheter, urinvägsbesvär, urinvägs besvär, urinvägsinfektion, urinvägs infektion, vattenkastningsbesvär, vattenkastnings besvär, vattenkastning, urinträngningar, urin trängningar, trängningar. trängningar till miktion, täta trängningar, UVI, UVI besvär, urinvägar, urin vägar, sveda vid miktion, sveda och täta trängningar, cystit, dysuri, inkontinens, pyelonefrit, frekventa miktioner, smärta vid miktion, miktionssmärta, miktions smärta | Micturition issues, micturition difficulties, urinary tract issues, urinary tract infection, issues with passing urine, passing urine, urinary urgency, urgency, frequent urgency, UTI, UTI issues, urinary tract, stinging during micturition, stinging and frequent urgency, cystitis, dysuria, incontinence, pyelonephritis, frequent micturition, pain during micturition, micturition pain, |
| **Cough/common cold/influenza** | Förkylning, förkyld, influensa, ÖLI, övre luftvägsinfektion, luftvägsinfektion, luftvägsbesvär, luftvägs besvär, luftvägar, långvarig hosta, hosta, hostar, slemhosta, slem hosta, torrhosta, torr hosta, rethosta, ret hosta, besvär med hosta. | Common cold, has common cold, influenza, URTI, upper respiratory tract infection, respiratory tract infection, respiratory tract issues, respiratory tracts, long lasting cough, cough, coughing, mucus cough, dry cough, hacking cough, issues with cough |
